# Goals of Care Conversations in Nursing Home and Assisted Living Care Plan Meetings

**DOI:** 10.1093/geroni/igab046.3609

**Published:** 2021-12-17

**Authors:** Gail Towsley, Djin Tay, Linda Edelman

**Affiliations:** 1 University of Utah College of Nursing, Salt Lake City, Utah, United States; 2 University of Utah, Salt Lake City, Utah, United States

## Abstract

Me & My Wishes is a novel systematic approach for long-term care residents living with dementia to record videos about their care preferences that can be shared with staff and families in care plan meetings. To understand how the videos were utilized in Goals of Care (GOC) conversations, we coded and analyzed transcripts of recorded care plan meetings at the time of sharing the video using a priori codes derived from GOC conversation elements. Coding discrepancies were resolved in team meetings; finalized codes were summarized to derive themes. Thirty-four care plan meeting conversations between residents (n=34), family members (n=29) and staff (n=35) were analyzed. Residents appreciated sharing personal histories and preferences via video, while staff members appreciated deeper understanding of residents’ care preferences. Two themes described care plan meeting conversations: Everyday Care - a checklist-style assessment of the resident’s daily care (e.g., help with activities of daily living), activities engaged in and satisfaction with care; and Clarifying Care Goals - checking the resident’s treatment preference (e.g., pain management, CPR), explaining hospice, or confirming the resident’s contact person. Several elements of GOC were not discussed (e.g., disease progression) and conversations lacked depth and comfort evidenced by apologetic language and abrupt transitions of topics rather than exploring alignment of goals with care preferences. Me & My Wishes videos are a mechanism for residents to voice preferences. Standardized guidance, which is lacking in long-term care, is needed to help care teams engage in meaningful conversations to ensure alignment of goals and treatment preferences.

